# Mutual Capacity Building to Reduce the Behavioral Health Treatment Gap Globally

**DOI:** 10.1007/s10488-019-00999-y

**Published:** 2019-12-10

**Authors:** Helen E. Jack, Bronwyn Myers, Kristen S. Regenauer, Jessica F. Magidson

**Affiliations:** 1 Department of Medicine, University of Washington, 1959 NE Pacific St, Seattle, WA 98195 USA; 2 Centre for Global Mental Health, Institute of Psychiatry, Psychology, and Neuroscience, King’s College London, London, UK; 3 Alcohol, Tobacco and Other Drug Research Unit, South African Medical Research Council, Francie van Zijl Drive, Parow, 7505 South Africa; 4 Department of Psychiatry and Mental Health, University of Cape Town, J-Block, Groote Schuur Hospital, Observatory, Cape Town, 7925 South Africa; 5 Department of Psychology, University of Maryland, 4094 Campus Drive, College Park, MD 20742 USA

**Keywords:** Global mental health, Peer support, Community health workers, Lay health workers, Mutual capacity building

## Abstract

Use of lay health workers for the treatment of common mental disorders is an expanding, yet still underutilized, opportunity for closing the behavioral health treatment gap globally. In this commentary, we describe how “mutual capacity building,” an equal exchange of ideas between low and middle-income countries (LMICs) and high-income countries (HICs) to promote shared learning, could promote the development and scale-up of therapies using lay health workers. We propose ways that task sharing models for behavioral health can inform and be supported by bidirectional learning across HICs and LMICs.

“Reverse innovation” has long been used to describe the adoption of ideas and technologies from low and middle-income countries (LMICs) in high-income countries (HICs). As HIC healthcare systems aim to reduce healthcare costs and expand access to care, they are increasingly looking to LMIC innovations (Bhattacharyya et al. 2017; Richards 2011). We prefer the term “mutual capacity building” (Binagwaho et al. 2013), as it acknowledges that resources, both material and intellectual, have long flowed from LMICs to HICs. The deep inequality between these settings cannot be ignored, and efforts to learn from LMICs should be collaborative and bidirectional.

Both HICs and LMICs face a large behavioral health treatment gap driven, in part, by severe global shortages of human resources for mental health (World Health Organization 2011). These insufficient resources are unevenly distributed, with most mental health professionals concentrated in urban areas and the private sector, leaving much of the population, and often the poorest and most vulnerable, without access to care. Unlike many other areas of service delivery, mental health care often does not require specific technology, but mainly relies on a trained workforce to provide treatment. HICs and LMICs face a mental health burden that requires similar, human resource-intensive treatment modalities for an effective response. Accordingly, development of strategies to increase human resources for mental health should be a priority for mutual capacity building.

Mental health care can be integrated in primary health services through “task sharing” initiatives, in which non-specialist health workers manage common mental disorders with supervision from specialist providers. Increasingly, those non-specialist workers are lay health workers (LHWs) in both HICs and LMICs. LHWs are people with minimal formal training who often share a diagnosis (peer provider) or community (community health worker, CHW) with the patients whom they treat. A recent systematic review described the characteristics of LHW interventions for behavioral health in both LMICs and HICs and found that the majority of interventions took place in LMICs (Barnett et al. 2018). The evidence-base on LHW interventions is growing, but they remain underutilized, particularly in HICs. Learning from interventions in LMICs, we highlight key lessons for working with LHWs in higher income settings along with opportunities for mutual capacity building (Binagwaho et al. 2013; Magidson et al. 2019a).

## Closing the Behavioral Health Treatment Gap: Task Sharing with Lay Health Workers

CHWs are typically from the community they serve, and therefore, bring cultural and community expertise through their familiarity with cultural practices, social structures, local social determinants of health, and community understandings of disease (Magidson et al. 2017). For instance, in Zimbabwe where there are only 14 psychiatrists for over 16 million people (Kidia et al. 2017), the Friendship Bench—an intervention in which CHWs deliver six-sessions of individual counselling for depression on benches outside of primary care clinics—was implemented (Chibanda et al. 2016). The Friendship Bench employs both cognitive behavioral therapy (CBT) techniques and CHWs’ knowledge of their community’s practices. A cluster randomized trial showed that it improved mental health outcomes for people with depression in poor, urban communities (Chibanda et al. 2016), and an adapted version of this intervention will be implemented in New York City (Rosenberg 2019). Similarly, CHWs have played a pivotal role in South Africa’s response to the HIV epidemic. Through training CHWs to conduct HIV testing and counseling (HTC), medication adherence support, and home-based care (Mottiar and Lodge 2018), South Africa is supporting the behavioral health needs of the largest antiretroviral treatment program globally. With a greater awareness of the relationship between substance use disorders (SUDs), mental health, and HIV outcomes, CHWs are now being trained to provide brief psychological treatments for SUDs and common mental disorders (Myers et al. 2019). Inspired by LMIC experiences, CHWs have been employed frequently in high-income countries (HICs) for chronic physical disease management (Jack et al. 2017). Although newer to HIC behavioral health services, early studies suggest promising behavioral health outcomes (Barnett et al. 2018).

One HIC response to the behavioral health treatment gap has been use of peer providers (Kent 2019); for instance, in SUD care, peer providers (i.e., peer recovery coaches or peer recovery specialists) are LHWs who are in recovery and support patients in harm reduction or substance use cessation (Jack et al. 2018). Outcomes of peer-delivered interventions in HICs are generally positive: for instance, a recent study on “recovery coaches” for patients with SUD showed a decrease in acute care visits and an increase in engagement in buprenorphine treatment (Magidson et al. 2019b). There is, however, an overall lack of rigorous studies, and results are difficult to generalize or replicate due to variability in peer role and training (Davidson et al. 1999; Eddie et al. 2019). Although peers are not uncommon in HICs, there is still considerable work needed to develop, test, and scale the role. Many questions remain about how to best integrate peers into the broader healthcare workforce and system for SUD and other mental health care (Kent 2019). While peers have long been part of behavioral health care in HICs (Davidson et al. 1999; Eddie et al. 2019; Kent 2019; Myrick and Del Vecchio 2016; Salzer et al. 2010), they are rarely employed in LMICs (Magidson et al. in press). As HICs continue to develop and standardize peer recovery models, LMICs could learn from and adapt models of task sharing that emphasize support from people who share a diagnosis, while HICs borrow and adapt strategies that LMICs have used to develop and scale-up CHW programs.

## Lessons from LMIC CHW Programs

There are several lessons from CHW interventions in LMICs that could contribute to mutual capacity building. First, LHWs may help mental health treatment programs develop a better understanding of culturally appropriate care and engage stigmatized groups. In South Africa, CHWs have expanded access for the maintenance of antiretroviral therapy and provided care with outcomes similar to physicians, possibly by making HIV care more acceptable for people who feel stigmatized and shamed by their disease (Myers et al. 2018). Second, LHWs could expand the coverage of services beyond the facilities where these services are generally provided, helping reach marginalized and vulnerable people. In LMICs, CHWs are used to deliver basic health services and health promotion messaging in patients’ homes and community spaces, bridging community and health service. Third, LHWs can be trained to provide evidence-based psychological therapies under the supervision and monitoring of providers with more specialised training. A recent systematic review found that LHW interventions in HICs typically use novel, community-based interventions, while those implemented in LMICs more frequently employ evidence-based treatments (Barnett et al. 2018). Studies in LMICs have shown that CHW-delivered brief treatments are feasible to deliver, acceptable to service users (Myers et al. 2019), and effective for improving behavioral health outcomes. However, LHWs require regular supervision, training, and monitoring to ensure continued fidelity to the intervention model (Magidson et al. 2017).

## Mutual Capacity Building to Address the Behavioral Health Treatment Gap

Adapting these lessons from LMICs is most ethically and effectively done collaboratively. Mutual capacity-building could take a variety of forms. More concretely, many HIC universities have contributed to or led research in LMICs, but these efforts could involve intervention testing and implementation in both settings. Funders can put out calls for mutual capacity building to address behavioral health problems that are high priority in both HICs and LMICs (DePasse and Lee 2013). These funding and research shifts, however, would necessitate breaking down conceptual, administrative, and departmental barriers that treat “global” health (in LMICs) and “community” health (in HICs) as distinct. Researchers from HICs and LMICs can collaborate to develop and strengthen frameworks for adapting interventions for a new setting (Wingood and DiClemente 2008) and rapidly testing their effectiveness, preparing the global mental health community to efficiently and effectively transfer evidence-based interventions across cultural lines.

## Conclusions

As the evidence on outcomes of LHW programs in LMICs expands and both LMICs and HICs continue to face a behavioral health treatment gap, now is the time for mutual capacity building. Mutual capacity building provides an opportunity to begin reshaping the power dynamics of global and community health research, making the research more collaborative, and providing a mechanism for elevating the voices of peers and community members in care delivery.
